# Indicators of complementary feeding and social vulnerability in the Brazilian Amazon

**DOI:** 10.11606/s1518-8787.2026060007513

**Published:** 2026-07-27

**Authors:** Steffany Martins Moreira, Isabel Giacomini, Lalucha Mazzucchetti, Marly Augusto Cardoso

**Affiliations:** I Universidade de São Paulo. Faculdade de Saúde Pública. Departamento de Nutrição. São Paulo, SP, Brasil; II Universidade de São Paulo. Faculdade de Saúde Pública. Programa de Pós-Graduação em Nutrição em Saúde Pública. São Paulo, SP, Brasil

**Keywords:** Complementary Feeding, Social Vulnerability, Child Health

## Abstract

**OBJECTIVE:**

To analyze Complementary Feeding Quality Indicators according to socioeconomic characteristics in Cruzeiro do Sul, Acre, Western Brazilian Amazon.

**METHODS:**

This is a cross-sectional analysis nested within the MINA - Brazil birth cohort (n = 971), using data from the 1-year (n = 774) and 2-year (n = 854) follow-up visits. Dietary intake was assessed using a 24-hour recall to construct the Complementary Feeding Quality Indicators, adapted from World Health Organization recommendations: minimum dietary diversity, meat and/or egg consumption, ultra-processed food consumption, and zero fruit and vegetable consumption . Socioeconomic, neonatal, and morbidity variables were analyzed according to a hierarchical conceptual model (distal, intermediate, and proximal levels). Prevalence ratios (PR) were estimated using Poisson regression with robust variance and multiple adjustment; variables were selected based on theoretical assumptions or an association with p < 0.10 at each level.

**RESULTS:**

Regarding meat and egg consumption, 79.0% of 1-year-old children and 89.9% of 2-year-old children consumed at least one food item from this group the previous day. High frequencies of ultra-processed food consumption were observed in the first (89.1%) and second (94.6%) years of life. The minimum dietary diversity indicator in the first year was less frequent among children from families with lower wealth indices (lowest tertile: PR = 0.74; 95%CI 0.63–0.86) and whose mothers were not engaged in paid work (PR = 0.87; 95%CI 0.80–0.95). Zero fruits and vegetables consumption was more prevalent among children from families with lower wealth indices (lowest tertilel: PR = 4.89; 95%CI 2.90–8.24).

**CONCLUSION:**

The results highlight the influence of socioeconomic factors on children’s dietary patterns, reinforcing the importance of intersectoral strategies to promote food and nutritional security.

## INTRODUCTION

Good nutrition in early life has the potential to ensure full child development, both cognitive and physical^1^. Given the importance of this period, the World Health Organization (WHO) recommends that breastfeeding be initiated within the first hour of life and maintained exclusively until six months of age. After this period, the gradual introduction of complementary feeding (CF) is recommended, including the introduction of semi-solid and solid foods, alongside the continued provision of breast milk until two years of age or older^2^.

Over the past few years, children’s nutritional status has been the subject of detailed analysis and global concern, highlighting the alarming situation of poor-quality child nutrition currently found worldwide. Between 2000 and 2022, a reduction in the global prevalence of stunting (low height-for-age) among children under five was observed, falling from 33% to approximately 22%. Despite this progress, the rate of decline remains insufficient to achieve the stunting rate target set for 2030 by the United Nations. Furthermore, the prevalence of wasting (low weight-for-height) and childhood overweight are also alarming, representing 6.8% and 5.6% of the global child population, respectively^3^. With the aim of guiding public health interventions and evaluating their impacts on children’s nutritional status, the WHO published, in 2021, a revised version of the Complementary Feeding Quality Indicators (CFQI) for children under two years of age. The document, which comprises 17 indicators, also enables standardized analysis of trends over time and the identification of populations at risk of malnutrition, providing important insights for the formulation of effective public policies^4^. Identifying factors associated with breastfeeding and complementary feeding practices is essential for a better interpretation of the proposed CFQIs. The available literature suggests that socioeconomic and demographic factors are related to the quality of child feeding^5,6^.

In Brazil, the Brazilian National Survey on Child Nutrition (ENANI - Estudo Nacional de Alimentação e Nutrição Infantil), a household-based survey, assessed complementary feeding practices among children under five years of age^7^. According to the data obtained, approximately 43% of Brazilian children aged six to 23 months did not meet the minimum dietary diversity (MDD) recommended by the WHO, and 22% had not consumed fruits or vegetables on the day prior to the interview^8^. Furthermore, although the consumption of ultra-processed foods is contraindicated before the age of two^9^, ENANI results indicated an 80% prevalence of consumption of this type of food on the previous day among children under two years of age residing in urban areas of Brazilian state capitals^10^.

In northern Brazil, particularly in remote areas and those bordering other countries, there is a scarcity of data on indicators of quality and determinants of child feeding practices. In these contexts, progress in child health indicators has been slower compared to other regions of the country^11^. Thus, children living in this region face unfavorable living and environmental conditions, making them even more vulnerable to the harmful effects of inadequate nutrition in early life.

Given the essential nature of feeding practices in early childhood, this study aims to analyze CFQIs according to socioeconomic characteristics in Cruzeiro do Sul (CZS), Acre, in the Brazilian Western Amazon.

## METHODS

### Setting, Study Design, and Target Population

This study conducted a cross-sectional analysis nested within the MINA-Brazil birth cohort, conducted in the municipality of CZS, Acre, in northern Brazil. CZS is located 636 km from the state capital, Rio Branco; it is the second most populous city in the state (with approximately 91,888 inhabitants) and has a Medium Human Development Index of 0.664, below the national average (0.786)^12^.

The MINA- Brazil cohort includes follow-up assessments of all children born between July^1^, 2015, and June 30, 2016, at the Hospital Estadual da Mulher e da Criança do Juruá, the only maternity hospital in CZS. Participants were recruited during pregnancy and after delivery at the maternity hospital. In total, 1,246 mother-infant pairs were considered eligible for follow-up in the subsequent phases of the study, as described in a previous publication^13^.

Mothers and their infants were followed up at 30–45 days via telephone interviews and at 6 months, 1 year, and 2 years via in-person interviews. After excluding 22 sets of twins, 1,224 mother-child pairs were considered eligible for this analysis; five children died by age two. At the 1 -year follow-up, 25 (2.0% of the 1,219 eligible) refused to participate, and 433 did not attend or could not be located, resulting in 774 children evaluated (63%). At 2 years, there were 41 refusals and 331 losses, with 854 children evaluated (70%). Compared to the baseline, the proportion (95%CI) of children from poorer families decreased from 24.9 (95%CI 22.5–27.4) to 19.3 (95%CI 16.8–22.1) at two years. The other characteristics—maternal education, primiparity, mode of delivery, child’s sex, low birth weight, and prematurity—were similar among participants retained and those lost to follow-up in each asseessment^13^.

The MINA-Braz il cohort received ethical approval from the Comitê de Ética em Pesquisa da Faculdade de Saúde Pública da Universidade de São Paulo, Brazil (protocols No. 872.613/2014 and No. 2.358.129/2017).

### Data Collection

In the maternity ward, after obtaining written consent to participate, a semi-structured questionnaire was administered to collect demographic information (mother’s age [categorized as < 19 years, 19–34 years, and ≥ 35 years], self-reported skin color of the mother [white, black, brown, yellow, and i ndigenous]) and family socioeconomic characteristics, such as maternal education (≤ 9 years, 10–12 years, > 12 years), being a Bolsa Família program beneficiary (yes or no), mother as the head of the household (yes or no), mother engaged in paid work (yes or no), and wealth index (calculated based on information regarding ownership of household goods using principal component analysis and divided into tertiles)^13,14^. Additional information on the mother’s clinical and obstetric history was extracted from the hospital medical records.

In the cohort follow-ups conducted during the child’s first and second years of life, questionnaires were administered regarding general health characteristics and childcare practices, in addition to blood collection. Blood hemoglobin (Hb) concentrations were measured using an ABX Micro 60 cell counter (Horiba, Montpellier, France), and serum retinol concentrations were assessed by high-performance liquid chromatography (HPLC). The cutoff points established by the WHO were adopted to define anemia (Hb < 110 g/L) and vitamin A deficiency (serum retinol concentrations < 1.05 μmol/L), as described in a previous publication^15^.

At the 1- and 2-year follow-ups, children’s food consumption was assessed using a 24-hour recall questionnaire covering the day prior to the interview, completed by the mother or primary caregiver, with interviews conducted by pre-trained interviewers, as described in a previous publication^16^. For each of the 23 food items /food groups asked about, as well as for the “other food” option, there were nine response categories regarding consumption (did not consume, upon waking, mid-morning, lunch, mid-afternoon, dinner, before bedtime, late at night, don’t know), with the option to select more than one choice^16^.

For the present analysis, we considered the 971 children assessed at the 1- and/or 2-year follow-ups . Of these, 774 and 854 provided complete dietary intake data at the 1 - and 2 -year follow-ups, respectively.

### Complementary Feeding Indicators

The outcome of interest was the adherence to four CFQIs proposed by the WHO in 2021 and adapted in this analysis, namely: i) MDD, ii) meat and/or egg consumption, iii) ultra-processed food consumption (UPF), and iv) zero fruit and vegetable consumption (ZFV)^4^. It was necessary to adapt some of the original indicators due to measurement limitations of the dietary survey and to better align with national guidelines on infant feeding. All indicators were constructed based on information collected regarding the child’s diet in the 24 hours prior to the interview.

- According to the WHO, the MDD refers to the consumption of at least five food items belonging to the eight food groups defined for the indicator: (1) breast milk; (2) grains, roots, and tubers; (3) legumes (beans, peas, lentils), nuts, and seeds; (4) dairy products; (5) meat products; (6) eggs; (7) fruits; (8) vegetables. The adaptation involved excluding the group of foods rich in vitamin A for which information was not available. Thus, this analysis considered the consumption of at least five of the following eight groups: (1) breast milk; (2) grains, roots, and tubers; (3) legumes (beans, peas, lentils), nuts, and seeds; (4) dairy products; (5) meat products; (6) eggs; (7) fruits; (8) vegetables. Although this modification limits direct comparability with studies using the original WHO classification, the structure of the indicator was maintained, preserving its ability to distinguish dietary diversity at the population level.
- The meat and/or egg consumption indicator corresponds to the consumption of at least one food item belonging to these groups.
- In its publication, the WHO proposes an “unhealthy foods” indicator, which assesses the intake of “sentinel foods” rich in sugar, fat, and salt that are more likely to be consumed by children, such as sweets and snacks, for example. However, in this context, homemade culinary preparations such as fruit-based sweets or recipes with vegetables and refined flours would fall under this indicator by definition. In this study, we chose to construct a specific indicator of UPF consumption, in line with national guidelines for child nutrition and based on the NOVA classification^9^, which considers the degree of food processing. This adaptation aims to increase the indicator’s specificity to capture processed foods associated with poorer health outcomes. The following were considered as UPF: industrialized yogurt and juice, soft drinks, sweets/candies, cookies, packaged savory snacks (chips), processed meats, instant noodles, chocolate-flavored milk drink, chocolate, fried savory snacks, ice cream, cheeseburgers, ham and cheese sandwiches, pizza, ham, condensed milk, cupuaçu cream dessert, ice pop, breakfast cereal, and sandwiches.
- The ZFV consumption indicator corresponds to the absence of fruit and vegetable intake on the previous day.

### Statistical Analysis

The frequencies of maternal and neonatal socioeconomic and demographic variables were analyzed for the 971 children with available data at 1 and/or 2 years of age. Next, the frequencies of feeding practices used to construct the CF indicators at the 1 - and 2 -year follow-up assessments were estimated. Prevalence ratios (PR) and 95% confidence intervals (95%CI) were obtained using Poisson regression with robust variance. A hierarchical conceptual model was developed for the initial selection of factors associated with the indicators in a multiple adjustment^17^. Starting from the distal level, socioeconomic and demographic data were used, followed by the intermediate level with neonatal variables and the proximal level with variables regarding childhood morbidities. At each level of determination, variables were selected based on theoretical assumptions or when associated with outcomes at each level of determination with p < 0.10.

Additionally, equiplot graphs were constructed for socioeconomic variables associated with the four CFQIs, with the aim of visualizing absolute inequalities between groups and facilitating the comparison of the magnitudes of differences between indicators and population strata^18^.

All analyses were performed using the statistical software Stata 18.0 (StataCorp LP, College Station, Texas, United States).

## RESULTS

Table 1 presents the socioeconomic and demographic characteristics of the mothers and newborns of the children participating in this study. The study population consisted mainly of mothers aged 19 to 34 years (73.1%), belonging to the top two-thirds of the wealth index (78.7%), and with 10 to 12 years of schooling (50.0%). Approximately one-third of the mothers were engaged in paid work (33.6%), and 38.7% received the Bolsa Família program benefit. Regarding neonatal characteristics, most children were born at a normal birth weight (86.9%), with 7.8% being preterm.

**Table 1:** Socioeconomic and demographic characteristics of the mothers and neonatal characteristics of the children assessed at 1- and 2-year follow-ups of the MINA-Brazil study (n = 971).

Variable	n^a^	%
Mother’s age		
< 19 years	162	16.7
19 to 34 years	710	73.1
≥ 35 years	100	10.2
Mother’s skin color (n = 952)		
White	119	12.5
Others^b^	833	87.5
Maternal education (n = 951)		
≤ 9 years	294	30.9
10 to 12 years	475	50.0
> 12 years	182	19.1
Bolsa Família beneficiary (n = 952)	368	38.7
Mother as head of household (n = 952)	141	14.8
Engaged in paid work (n = 952)	320	33.6
Wealth index (n = 952)		
1st tertile (lowest)	203	21.3
2nd tertile	347	36.5
3rd tertile (largest)	402	42.2
Number of antenatal care visits		
< 6 visits	196	20.3
≥ 6 visits	769	79.7
Type of delivery		
Normal	509	52.4
Cesarean	462	47.6
Birth weight (n = 970)		
< 2,500 g	64	6.6
≥ 2,500 g to 3,999 g	843	86.9
≥ 4,000 g	63	6.5
Prematurity	76	7.8

^a^ Totals for each variable differ due to missing data.

^b^ Refers to people: Black (3.36%), Brown (78.36%), Indigenous (0.84%), or Yellow (4.94%).

Table 2 presents the consumption frequencies of the food groups used in the composition of the dietary diversity indicators analyzed. A high frequency of consumption of UPF was observed in both follow-ups (>85%), in addition to changes in the CFQI between one and two years. There was a reduction in the MDD (from 71.4% to 64.0%) and an increase in the consumption of meat and/or eggs (from 78.0% to 90.5%). On the other hand, the absence of fruit and vegetable consumption increased during the period (from 12.9% to 16.5%).

**Table 2:** Frequencies of components of complementary feeding indicators in the one - and two-year follow-ups of the MINA-Brazil study (n = 972). Cruzeiro do Sul, Acre, 2018.

Child feeding indicators	One year (n = 774)		Two years (n = 854)	
	n^a^	%	n^a^	%
Minimum dietary diversity (consumption of at least five of the eight food groups)	549	71.4	531	64.0
Consumption of at least one food from a food group	9	1.2	13	1.6
Consumption of at least one food from two food groups	27	3.5	27	3.3
Consumption of at least one food from three food groups	85	11.0	93	11.2
Consumption of at least one food item from four food groups	98	12.8	165	19.9
Consumption of at least one food from five food groups	175	22.8	244	29.4
Consumption of at least one food item from six food groups	206	26.8	202	24.4
Consumption of at least one food item from seven food groups	143	18.6	76	9.2
Consumption of at least one food item from eight food groups	25	3.2	9	1.1
Meat and/or egg consumption	604	78.0	773	90.5
Consumption of any ultra-processed food	676	87.3	790	92.8
Consumption of at least one food item from the following categories: crackers, cookies, sweets, and cake	549	71.0	633	74.4
Consumption of at least one food item from the processed meats group	38	4.9	86	10.1
Consumption of at least one food item from the instant noodles, packaged snacks, or fried snacks group	192	24.8	249	29.3
Consumption of at least one food item from the sweetened beverages group	472	61.0	584	68.6
Zero fruit and vegetable consumption	100	12.9	138	16.5

^a^ For each indicator of child nutrition, the totals in each category may differ due to missing data.

Adherence to the MDD indicator in the first year of life was lower among children from families in the lowest third of the wealth index and among those whose mothers were not engaged in paid work (Table 3). At two years of age, the lowest frequency of MDD was observed among children of mothers with lower educational attainment and heads of households. Lower adherence to the meat and/or egg indicator was observed among children of mothers without paid employment, in both the first and second years (Table 4). Similar inequalities were identified for the ZFV indicator, which was more frequent among children from poorer families (Table 4).

**Table 3:** Adjusted prevalence ratio and respective 95% confidence intervals for factors associated with indicators of minimum dietary diversity in the one- and two-year follow-ups of the MINA-Brazil study.

Variable	MDD One year (n = 767)^a^	MDD Two years (n = 829)^a^
	PR (95%CI)	PR (95%CI)
Wealth tertile		
1st tertile (lowest)	0.74 (0.63–0.86)	-
2nd tertile	0.90 (0.82–0.99)	-
3rd tertile (highest)	Ref.	-
Mother’s skin color		
White	Ref.	-
Others^c^	0.89 (0.80–1.00)	-
Maternal education		
≤ 9 years	-	0.71 (0.60–0.83)^b^
Between 10 and 12 years	-	0.84 (0.75–0.94)^b^
> 12 years	-	Ref.
Bolsa Família beneficiary		
No	-	1.11 (0.98–1.26)
Yes	-	Ref.
Mother as head of household		
No	-	Ref.
Yes	-	0.77 (0.64–0.92)^b^
Engages in paid work		
No	0.87 (0.80–0.95)^b^	-
Yes	Ref.	

PR: prevalence ratio; 95%CI: 95% confidence interval; MDD: minimum dietary diversity.

^a^ Totals for each variable differ due to missing data.

^b^ p < 0.05.

^c^ Refers to people: Black, Brown, Indigenous or Yellow.

**Table 4:** Adjusted prevalence ratio and respective 95% confidence intervals for factors associated with meat and/or egg consumption and zero consumption of fruits and vegetables in the one - and two -year follow-ups of the MINA-Brazil study.

Variable	Consumption of meat and/or eggs One year (n = 773)^a^	Consumption of meat and/or eggs Two years (n = 851)^a^
	PR (95%CI)	PR (95%CI)
Engaged in paid work		
No	0.88 (0.81–0.97)	0.93 (0.88–0.97)^b^
Yes	Ref.	Ref.
Vitamin A deficiency - child		
No	Ref.	
Yes	0.80 (0.64–0.99)	
**Variable**	**ZFV consumption One year (n = 761)^a^**	**ZFV consumption Two years (n = 732)^a^**
	**PR (95%CI)**	**PR (95%CI)**
Maternal education		
≤ 9 years	-	1.56 (0.93–2.60)
Between 10 and 12 years	-	0.99 (0.60–1.63)
> 12 years	-	Ref.
Wealth index		
1st tertile (lowest)	4.89 (2.90–8.24)^b^	-
2nd tertile	2.47 (1.45–4.21)^b^	-
3rd tertile (highest)	Ref.	-

PR: prevalence ratio; 95%CI: 95% confidence interval; ZFV: zero fruit and vegetable consumption.

^a^ Totals for each variable differ due to missing data.

^b^ p < 0.05.

Regarding UPF consumption, children of mothers with higher educational attainment (> 12 years) had lower consumption frequencies, while those whose mothers had lower educational attainment had higher consumption, both at one and two years of age (Table 5).

**Table 5:** Adjusted prevalence ratio and respective 95% confidence intervals for factors associated with consumption of ultra-processed foods at one- and two-year follow-ups of the MINA-Brazil study.

Variable	UPF consumption One year (n = 768)^a^	UPF consumption Two years (n = 829)^a^
	PR (95%CI)	PR (95%CI)
Mother’s age		
< 19 years	1.14 (0.98–1.32)	1.07 (0.99–1.16)
19 to 34 years	1.07 (0.92–1.24)	1.04 (0.95–1.13)
≥ 35 years	Ref.	Ref.
Mother’s skin color		
White	Ref.	-
Others^c^	0.93 (0.85–1.01)	-
Maternal education		
≤ 9 years	1.12 (0.98–1.27)	1.11 (1.04–1.20)^b^
Between 10 and 12 years	1.13 (1.01–1.27)^b^	1.09 (1.02–1.17)^b^
> 12 years	Ref.	Ref.
Wealth index		
1st tertile (lowest)	0.94 (0.85–1.04)	-
2nd tertile	1.02 (0.95–1.10)	-
3rd tertile (highest)	Ref.	-
Child’s birth weight (n = 970)		
< 2,500 g	-	0.95 (0.87–1.04)
≥ 2,500 g to 3,999 g	-	Ref.
≥ 4,000 g	-	1.04 (0.99–1.09)
Anemia		
No	Ref.	-
Yes	1.08 (1.02–1.15)^b^	-
Vitamin A deficiency		
No	Ref.	-
Yes	1.10 (1.03–1.17)^b^	-

PR: prevalence ratio; 95%CI: 95% confidence interval; UPF: ultra-processed foods.

^a^ Totals for each variable differ due to missing data.

^b^ p < 0.05.

^c^ Refers to people: Black, Brown, Indigenous or Yellow.

The Figure shows consistent differences in CFQIs according to wealth index and maternal education level in both follow-ups. For MDD, higher frequencies are observed among children from families with better socioeconomic conditions, both in terms of wealth and maternal education, with a similar pattern in both follow-ups. A similar trend was observed for the consumption of meat and/or eggs, with higher frequencies among children from more affluent families. In contrast, the consumption of UPF was high across all strata, although slightly lower among children of mothers with higher educational attainment. On the other hand, the ZFV indicator was more frequent among children from families with poorer socioeconomic conditions, highlighting unfavorable inequalities in these groups. Overall, the equiplots indicate differences in children’s dietary patterns according to socioeconomic conditions, which are maintained over time.

**Figure:**
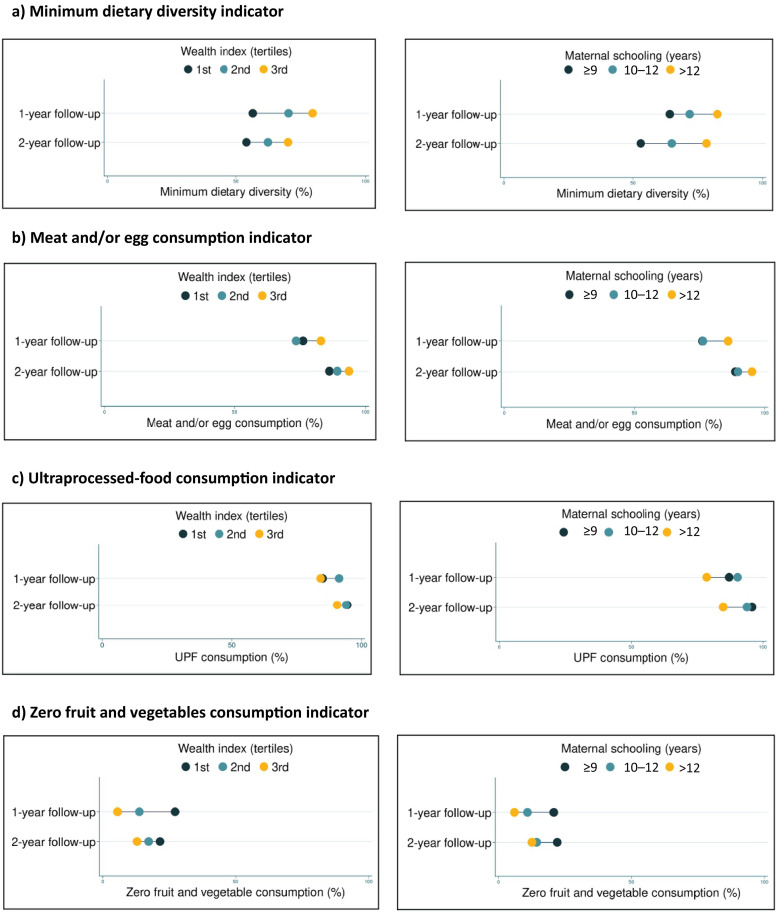
Equiplot plots of four indicators of complementary feeding quality studied according to tertiles of the wealth index and maternal education in the 1- and 2-year follow-ups of the MINA- Brazil cohort.

## DISCUSSION

The results of this study showed that positive indicators of CF (MDD and meat and/or eggs consumption were more frequent among children from more favorable socioeconomic backgrounds, while lower frequencies were observed among children from poorer families and whose mothers had lower levels of education, were not engaged in paid work, or were heads of the household. Similarly, negative indicators of dietary diversity (consumption of UPF and ZFV) were more frequent among children from lower-income families and whose mothers had lower levels of education.

The influence of socioeconomic characteristics on the diversity of children’s diets has been observed in various studies, indicating that families with higher income and greater purchasing power have greater access to different food groups and, therefore, provide a more diverse diet to children^5,19,20^. Although not all studies find an association between family income and the consumption of meat and/or eggs^21^, national evidence indicates higher consumption of fresh foods, including unprocessed meats, among children from families with greater purchasing power^22^. These findings are consistent with the results of the present study and suggest that the absence of maternal income may contribute to greater economic vulnerability in the household.

Data from ENANI 2019 indicated that the Northern region of Brazil had the lowest prevalence of MDD among children aged 6 to 23 months (54.8%) compared to the national average (63.4%)^10^. A lower prevalence of this indicator was also observed among children whose mothers or caregivers had low educational attainment (zero to seven years of schooling: 50.6%) and among those living in households with moderate or severe food insecurity (52.6%). These findings reinforce the relationship between adverse socioeconomic conditions and poorer dietary patterns in childhood, consistent with the results observed here.

It was observed that the higher the maternal education level, the lower the children’s consumption of UPF. This pattern is consistent with literature and may reflect socioeconomic differences that influence access, knowledge, and dietary choices. In a cohort study conducted in the state of Maranhão (n = 1,185), children whose mothers had less than eight years of schooling were 37% more likely to consume UPF; among those with nine to 11 years of schooling, the likelihood was 25% higher, compared to children of mothers with more than 12 years of schooling^23^. Similar results were observed in southern Brazil in a cross-sectional study conducted at a tertiary hospital in Porto Alegre (n = 300) between 2012 and 2013, showing an inverse association between maternal education and UPF consumption among children aged 4 to 24 months^24^. Consistent results were identified by ENANI 2019, which reported lower UPF consumption among children whose mothers had higher levels of education, compared to those with less than seven years of schooling or between eight and 11 years of schooling^25^. This pattern may reflect greater health literacy among mothers with higher levels of education, favoring healthier dietary choices during childhood^26^.

In the present study, the ZFV indicator was more frequent among children from lower-income families, indicating a less favorable dietary pattern in these groups. This finding is consistent with low consumption rates in the Brazilian population^27^, which show reduced consumption of these foods among populations with lower socioeconomic status, possibly due to limited access and the higher cost of fresh foods. Given the importance of fruits and vegetables for diet quality and for establishing healthy eating habits in childhood^28^, the lack of consumption of these foods may have implications for health in later stages of life^30^.

Among the limitations of this study, it is worth noting that adjustments to the operationalization of the CF indicators prevented full adherence to the WHO proposal. In particular, the lack of information on foods rich in vitamin A prevented their inclusion in the minimum dietary diversity indicator, which may limit comparability with other studies, although the overall structure was preserved. The 24-hour recall is subject to measurement error and memory bias and may not reflect individual habitual consumption; furthermore, it was not possible to adjust for intra-individual variability, given the use of measurements at different time points (one- and two- year follow-ups). The UPF indicator was based on the NOVA classification, which may limit comparisons with the WHO indicator, although it increases specificity for foods associated with worse outcomes. The analyses are cross-sectional in nature, limiting causal inference, and follow-up losses may have introduced selection bias, although there is little evidence of relevant differences between participants who were followed up and those who were not.

The findings of this study point to important differences in CF patterns among children in the Western Brazilian Amazon, reflecting the impact of socioeconomic conditions on the quality of children’s diets. These results reinforce the need for intersectoral strategies aimed at promoting healthy eating in childhood to reduce inequalities and foster healthier food environments.

## Data Availability

Data is available upon request to the corresponding author.
