# Supplementary material for: Indicators of complementary feeding and social vulnerability in the Brazilian Amazon
Source: Rev Saude Publica. 2026 Jul 27;60:e34. doi: 10.11606/s1518-8787.2026060007513 (PMC13405610; doi:10.11606/s1518-8787.2026060007513)
Supplement: Translation

# Translation: Indicadores da alimentação complementar e vulnerabilidade social na Amazônia brasileira

**Authors:** Steffany Martins Moreira, Isabel Giacomini, Lalucha Mazzucchetti, Marly Augusto Cardoso

**Affiliations:** I Universidade de São Paulo. Faculdade de Saúde Pública. Departamento de Nutrição. São Paulo, SP, Brasil; II Universidade de São Paulo. Faculdade de Saúde Pública. Programa de Pós-Graduação em Nutrição em Saúde Pública. São Paulo, SP, Brasil

**Keywords:** Alimentação Complementar, Vulnerabilidade Social, Saúde Infantil

## Abstract

**OBJETIVO:**

Analisar os indicadores de qualidade da alimentação complementar infantil segundo características socioeconômicas em Cruzeiro do Sul, Acre, Amazônia Ocidental Brasileira.

**MÉTODOS:**

Trata-se de análise transversal aninhada à coorte de nascimentos MINA-Brasil (n = 971), com dados dos seguimentos de um (n = 774) e dois (n = 854) anos. O consumo alimentar foi avaliado por recordatório de 24 horas para construção dos indicadores de qualidade da alimentação complementar infantil, adaptados das recomendações da Organização Mundial da Saúde: diversidade mínima alimentar, consumo de carnes e/ou ovos, consumo de alimentos ultraprocessados e zero consumo de frutas e vegetais. Variáveis socioeconômicas, neonatais e de morbidades foram analisadas segundo modelo conceitual hierárquico (níveis distal, intermediário e proximal). As associações foram estimadas por regressão de Poisson com variância robusta e ajuste múltiplo; variáveis foram selecionadas com base em pressupostos teóricos ou associação com p < 0,10 em cada nível.

**RESULTADOS:**

Quanto ao consumo de carnes/ovos, 79,0% das crianças de um ano e 89,9% das crianças de dois anos consumiram ao menos um alimento desse grupo no dia anterior. Altas frequências de alimentos ultraprocessados foram observadas no primeiro (89,1%) e no segundo ano (94,6%). O indicador diversidade mínima alimentar no primeiro ano foi menos frequente entre crianças de famílias com menores índices de riqueza (menor terço: RP = 0,74; IC95% 0,63–0,86) e cujas mães não exerciam atividade remunerada (RP = 0,87; IC95% 0,80–0,95). O zero consumo de frutas e vegetais foi mais prevalente entre famílias com menor riqueza (menor terço: RP = 4,89; IC95% 2,90–8,24).

**CONCLUSÃO:**

Os resultados evidenciam a influência dos fatores socioeconômicos sobre os padrões alimentares infantis, reforçando a importância de estratégias intersetoriais para promoção da segurança alimentar e nutricional.

## INTRODUÇÃO

Uma boa nutrição no início da vida tem o potencial de proporcionar um desenvolvimento infantil pleno, tanto cognitivo como físico^1^. Devido a importância desse período, a Organização Mundial da Saúde (OMS) preconiza que a amamentação seja iniciada na primeira hora de vida e mantida exclusivamente até os seis meses de idade. Após esse período, recomenda-se a introdução gradual da alimentação complementar (AC), com introdução de alimentos semissólidos e sólidos, em concomitância com o oferecimento do leite materno até os dois anos de idade ou mais^2^.

Ao longo dos últimos anos, o estado nutricional infantil têm sido objeto de análise detalhada e preocupação global, evidenciando o cenário alarmante da má qualidade da alimentação infantil encontrado no mundo atualmente. Entre os anos 2000 e 2022, observou-se uma redução na prevalência global de *stunting* (déficit de altura para idade) entre crianças menores de cinco anos, passando de 33% para aproximadamente 22%. Apesar desse progresso, o ritmo de declínio ainda se mostra insuficiente para que a meta da taxa de retardo no crescimento estabelecida para 2030 pelas Organização das Nações Unidas seja alcançada. Além disso, as prevalências de *wasting* (déficit de peso para altura) e excesso de peso infantil também são alarmantes, representando 6,8% e 5,6% da população global infantil, respectivamente^3^. Com o objetivo de orientar intervenções em saúde pública e avaliar seus impactos sobre o estado nutricional infantil, a OMS publicou, em 2021, uma versão revisada dos Indicadores de Qualidade da Alimentação Complementar (IQAC) para crianças menores de dois anos. O documento, que reúne 17 indicadores, também permite a análise padronizada de tendências ao longo do tempo e a identificação de populações em risco de má nutrição, oferecendo subsídios importantes para a formulação de políticas públicas efetivas^4^. A identificação de fatores associados às práticas de amamentação e AC é essencial para uma melhor interpretação dos IQAC propostos. A literatura disponível sugere que fatores socioeconômicos e demográficos estão relacionados com a qualidade da alimentação infantil^5,6^.

No Brasil, o Estudo Nacional de Alimentação e Nutrição Infantil (Enani), um inquérito de base domiciliar, avaliou as práticas de AC entre crianças menores de cinco anos^7^. Segundo os dados obtidos, aproximadamente 43% das crianças brasileiras com idade entre 6 e 23 meses não atingiram a diversidade mínima alimentar recomendada pela OMS e 22% não haviam consumido frutas ou hortaliças no dia anterior à entrevista^8^. Além disso, embora o consumo de alimentos ultraprocessados seja contraindicado antes dos dois anos de idade^9^, os resultados do ENANI indicaram uma prevalência de 80% do consumo desse tipo de alimento no dia anterior entre crianças menores de dois anos residentes em áreas urbanas das capitais brasileiras^10^.

Na região norte do Brasil, sobretudo em áreas remotas e de fronteira com outros países, há escassez de dados sobre indicadores de qualidade e determinantes das práticas de AC. Nesses contextos, o avanço dos indicadores de saúde infantil tem ocorrido de forma mais lenta quando comparado a outras regiões do país^11^. Assim, as crianças que vivem nessa região enfrentam condições de vida e ambientais desfavoráveis, tornando-as ainda mais vulneráveis aos efeitos prejudiciais da nutrição inadequada no início da vida.

Diante da essencialidade das práticas alimentares na primeira infância, este trabalho visa analisar os IQAC segundo características socioeconômicas em Cruzeiro do Sul, Acre, Amazônia Ocidental Brasileira.

## MÉTODOS

### Área, Delineamento do Estudo e População-Alvo

Este estudo realizou análise transversal aninhada à coorte de nascimentos MINA utilizou dados da coorte de nascimentos MINA-Brasil - Saúde e Nutrição Materno-Infantil no Acre, realizada no município de Cruzeiro do Sul (CZS), Acre, na região Norte do Brasil. CZS distancia-se 636 km da capital do estado, Rio Branco, é a segunda cidade mais populosa do estado (com aproximadamente 91.888 habitantes) e possui um Índice de Desenvolvimento Humano Médio de 0,664, abaixo da média nacional (0,786)^12^.

A coorte MINA-Brasil, abrange o acompanhamento de todas as crianças nascidas no período de 1^o^ de julho de 2015 a 30 de junho de 2016 no Hospital Estadual da Mulher e da Criança do Juruá, única maternidade de CZS. O recrutamento das participantes ocorreu durante a gravidez e após o parto na maternidade. No total, 1.246 pares de mãe-bebê foram considerados elegíveis para acompanhamento nas fases seguintes do estudo, conforme descrito em publicação anterior^13^.

As mães e seus bebês foram acompanhados aos 30–45 dias por entrevistas telefônicas e aos seis meses, um e dois anos por entrevistas presenciais. Após a exclusão de 22 gêmeos, 1.224 pares mãe-filho foram considerados elegíveis para esta análise; cinco crianças morreram até os dois anos. No seguimento de um ano, 25 (2,0% dos 1.219 elegíveis) recusaram participação e 433 não compareceram ou não foram localizados, resultando em 774 crianças avaliadas (63%). Aos dois anos, houve 41 recusas e 331 perdas, com 854 crianças avaliadas (70%). Em relação à linha de base, a proporção (IC95%) de crianças de famílias mais pobres diminuiu de 24,9 (IC95% 22,5–27,4) para 19,3 (IC95% 16,8–22,1) aos dois anos. As demais características – escolaridade materna, primiparidade, tipo de parto, sexo da criança, baixo peso ao nascer e prematuridade – foram semelhantes entre participantes acompanhados e perdidos em cada onda^13^.

A coorte MINA-Brasil obteve aprovação ética pelo Comitê de Ética em Pesquisa da Faculdade de Saúde Pública da Universidade de São Paulo, Brasil (protocolos nº 872.613/2014 e nº 2.358.129/2017).

### Coleta de Dados

Na maternidade, após consentimento à participação por escrito, foi aplicado um questionário semiestruturado, por meio do qual foram coletadas informações demográficas (idade da mãe [categorizada em < 19 anos, 19–34 anos e ≥ 35 anos], cor da pele autor referida da mãe [branca, preta, parda, amarela e indígena]) e socioeconômicas da família, tais como escolaridade materna (≤ 9 anos, 10–12 anos, > 12 anos), recebimento de benefício do Programa Bolsa Família (sim ou não), mãe chefe de domicílio (sim ou não), mãe realiza atividade remunerada (sim ou não) e índice de riqueza (calculado a partir das informações de posse de bens domésticos por meio da análise de componentes principais e dividido em terços)^13,14^. Outras informações sobre o histórico clínico e obstétrico da mãe foram extraídas do prontuário hospitalar.

Nos seguimentos da coorte realizados no primeiro e no segundo ano de vida da criança, foram aplicados questionários sobre dados referentes a características gerais de saúde e de práticas de AC da criança, além de coleta de sangue. As concentrações de hemoglobina sanguínea (Hb) foram medidas por contador de células ABX Micro 60 (Horiba, Montpellier, França) e as concentrações séricas de retinol foram avaliadas por cromatografia líquida de alta performance (HPLC). Os pontos de corte estabelecidos pela OMS foram adotados para definir anemia (Hb < 110 g/L) e insuficiência de vitamina A (concentrações séricas de retinol < 1,05 μmol/L), conforme descrição em publicação anterior^15^.

Nos seguimentos de um e dois anos de idade, o consumo alimentar das crianças foi avaliado por meio de um inquérito recordatório de 24 horas, referente ao dia anterior à entrevista, respondido pela mãe ou cuidador principal, com entrevistas conduzidas por entrevistadores previamente treinados, conforme descrito em publicação anterior^16^. Para cada um dos 23 alimentos/grupos de alimentos questionados, e para a opção “outro alimento”, havia nove categorias de respostas (não consumiu, ao acordar, meio da manhã, almoço, meio da tarde, jantar, antes de dormir, madrugada, não sabe), sendo possível selecionar mais de uma opção^16^.

Para a presente análise, considerou-se as 971 crianças acompanhadas no seguimento de um e/ou dois anos de idade. Destas, 774 e 854 completaram dados de consumo alimentar nos seguimentos de um e dois anos, respectivamente.

### Indicadores de Alimentação Complementar

O desfecho de interesse foi a adesão a quatro IQAC propostos pela OMS em 2021 e adaptados nesta análise, sendo eles: i) diversidade mínima alimentar (DMA), ii) consumo de carnes e/ou ovos, iii) consumo de alimentos ultraprocessados (AUP) e iv) zero consumo de frutas e vegetais (ZFV)^4^. As adaptações foram realizadas devido a limitações de mensuração do inquérito alimentar e para melhor alinhamento às diretrizes nacionais de alimentação infantil. Todos os indicadores foram construídos a partir das informações coletadas sobre a alimentação da criança nas 24 horas anteriores à entrevista.

i) De acordo com a OMS, a DMA diz respeito ao consumo de ao menos cinco alimentos pertencentes aos oito grupos alimentares definidos para o indicador: (1) leite materno; (2) grãos, raízes e tubérculos; (3) leguminosas (feijão, ervilha, lentilha), nozes e sementes; (4) laticínios; (5) alimentos cárneos; (6) ovos; (7) frutas; (8) legumes e verduras. A adaptação consistiu na exclusão do grupo de alimentos ricos em vitamina A cuja informação não estava disponível. Dessa forma, considerou-se nesta análise o consumo de pelo menos cinco dos oito grupos a seguir: (1) leite materno; (2) grãos, raízes e tubérculos; (3) leguminosas (feijão, ervilha, lentilha), nozes e sementes; (4) laticínios; (5) alimentos cárneos; (6) ovos; (7) frutas; (8) legumes e verduras. Embora essa modificação limite a comparabilidade direta com estudos que utilizam a classificação original da OMS, a estrutura do indicador foi mantida, preservando sua capacidade de discriminar a diversidade alimentar em nível populacional.

ii) O indicador de consumo de alimentos cárneos e/ou ovos corresponde ao consumo de pelo menos um alimento pertencente a esses grupos.

iii) Em sua publicação, a OMS propõe um indicador de “alimentos não saudáveis”, que avalia a ingestão de “alimentos sentinelas” ricos em açúcar, gordura e sal mais propensos a serem consumidos por crianças, como doces e salgadinhos, por exemplo. Contudo, nesse contexto, preparações culinárias caseiras como doces à base de frutas ou receitas com hortaliças e farinhas refinadas se enquadrariam nesse indicador por definição. Neste estudo, optou-se por construir um indicador específico de consumo de AUP, em consonância com as diretrizes nacionais de alimentação infantil e com base na classificação NOVA^9^, que considera o grau de processamento dos alimentos. Essa adaptação visa aumentar a especificidade do indicador para captar alimentos industrializados associados a piores desfechos de saúde. Foram considerados como AUP: iogurte e suco industrializados, refrigerantes, guloseimas, biscoito, salgadinho, embutidos, macarrão instantâneo, achocolatado, chocolate, salgados fritos, sorvete, x-salada, misto, pizza, presunto, leite condensado, creme de cupuaçu, geladinho, cereal matinal e sanduíche.

iv) O indicador zero consumo de frutas e vegetais corresponde à ausência de consumo de frutas, legumes e verduras no dia anterior.

### Análise Estatística

As frequências das variáveis socioeconômicas e demográficas maternas e neonatais foram analisadas para as 971 crianças com dados disponíveis aos um e/ou dois anos de idade. Em seguida, foram estimadas as frequências das práticas alimentares utilizadas na construção dos indicadores de AC nos seguimentos de um e dois anos. A razão de prevalência (RP) e intervalos com 95% de confiança (IC95%) foram obtidos por meio da regressão de Poisson com variância robusta. Elaborou-se modelo conceitual hierárquico para seleção inicial dos fatores associados aos indicadores em ajuste múltiplo^17^. A partir do nível distal, foram utilizados dados socioeconômicos e demográficos, seguindo-se o nível intermediário com variáveis neonatais e nível proximal com as variáveis sobre morbidades da criança. Em cada nível de determinação, foram selecionadas as variáveis segundo pressupostos teóricos ou quando associadas aos desfechos em cada nível de determinação com p < 0,10.

Adicionalmente, foram construídos gráficos do tipo *equiplot* para variáveis socioeconômicas associadas aos quatro IQAC, com o objetivo de visualizar desigualdades absolutas entre grupos e facilitar a comparação das magnitudes das diferenças entre indicadores e estratos populacionais^18^.

Todas as análises foram realizadas com auxílio do *software* estatístico Stata 18.0 (StataCorp LP, College Station, Texas, Estados Unidos).

## RESULTADOS

A Tabela 1 apresenta as características socioeconômicas e demográficas das mães e neonatais das crianças participantes deste estudo. A população de estudo foi composta majoritariamente por mães com idade entre 19 e 34 anos (73,1%), pertencentes aos dois terços superiores do índice de riqueza (78,7%) e com escolaridade entre 10 e 12 anos (50,0%). Aproximadamente um terço das mães realizava atividade remunerada (33,6%) e 38,7% recebiam o benefício do Bolsa Família. Quanto às características neonatais, a maioria das crianças nasceu com peso adequado (86,9%), sendo 7,8% prematuras.

**Tabela 1:** Características socioeconômicas e demográficas das mães e neonatais das crianças avaliadas nos seguimentos de um e dois anos de idade no Estudo MINA-Brasil (n = 971).

Variável	n^a^	%
Idade materna		
< 19 anos	162	16,7
19 a 34 anos	710	73,1
≥ 35 anos	100	10,2
Cor da pele da mãe (n = 952)		
Branca	119	12,5
Outras^b^	833	87,5
Escolaridade materna (n = 951)		
≤ 9 anos	294	30,9
10 a 12 anos	475	50,0
> 12 anos	182	19,1
Recebe Bolsa Família (n = 952)	368	38,7
Mãe chefe de domicílio (n = 952)	141	14,8
Realiza atividade remunerada (n = 952)	320	33,6
Índice de riqueza (n = 952)		
1º terço (menor)	203	21,3
2º terço	347	36,5
3º terço (maior)	402	42,2
Tipo de parto		
Normal	509	52,4
Cesárea	462	47,6
Peso da criança ao nascer (n = 970)		
< 2.500 g	64	6,6
≥ 2.500 g a 3.999 g	843	86,9
≥ 4.000 g	63	6,5
Prematuridade	76	7,8

^a^ Totais em cada variável diferem devido à ausência de informação.

^b^ Refere-se a pessoas: pretas (3,36%), pardas (78,36%), indígenas (0,84%) ou amarelas (4,94%).

A Tabela 2 apresenta as frequências de consumo dos grupos de alimentos utilizados na composição dos indicadores de AC analisados. Observou-se elevada frequência de consumo de AUP em ambos os seguimentos (> 85%), além de mudanças nos IQAC entre um e dois anos. Houve redução na diversidade mínima da alimentação (de 71,4% para 64,0%) e aumento no consumo de carnes e/ou ovos (de 78,0% para 90,5%). Por outro lado, a ausência de consumo de frutas, legumes e verduras aumentou no período (de 12,9% para 16,5%).

**Tabela 2:** Frequências de componentes dos indicadores da alimentação complementar nos seguimentos de um e dois anos do Estudo MINA-Brasil (n = 972). Cruzeiro do Sul, Acre, 2018.

Indicadores da alimentação infantil	Um ano (n = 774)		Dois anos (n = 854)	
	n^a^	%	n^a^	%
Diversidade mínima alimentar (consumo de ao menos cinco dos oito grupos alimentares)	549	71,4	531	64,0
Consumo de pelo menos um alimento de um grupo alimentar	9	1,2	13	1,6
Consumo de pelo menos um alimento de dois grupos alimentares	27	3,5	27	3,3
Consumo de pelo menos um alimento de três grupos alimentares	85	11,0	93	11,2
Consumo de pelo menos um alimento de quatro grupos alimentares	98	12,8	165	19,9
Consumo de pelo menos um alimento de cinco grupos alimentares	175	22,8	244	29,4
Consumo de pelo menos um alimento de seis grupos alimentares	206	26,8	202	24,4
Consumo de pelo menos um alimento de sete grupos alimentares	143	18,6	76	9,2
Consumo de pelo menos um alimento de oito grupos alimentares	25	3,2	9	1,1
Consumo de carnes e/ou ovos	604	78,0	773	90,5
Consumo de algum alimento ultraprocessado	676	87,3	790	92,8
Consumo de pelo menos um alimento de bolacha, biscoito, guloseimas e bolo	549	71,0	633	74,4
Consumo de pelo menos um alimento do grupo dos embutidos	38	4,9	86	10,1
Consumo de pelo menos um alimento do grupo de macarrão instantâneo, salgadinho de pacote, salgadinho frito	192	24,8	249	29,3
Consumo de pelo menos um alimento do grupo das bebidas adoçadas	472	61,0	584	68,6
Consumo zero de frutas e vegetais	100	12,9	138	16,5

^a^ Em cada indicador da alimentação infantil, os totais em cada seguimento podem diferir devido à ausência de informações.

A adesão ao indicador DMA no primeiro ano de vida foi menor entre crianças de famílias pertencentes ao menor terço do índice de riqueza e entre aquelas cujas mães não exerciam atividade remunerada (Tabela 3). Aos dois anos, a menor frequência de DMA foi observada entre filhos de mães com menor escolaridade e chefes de domicílio. A menor adesão ao consumo de carnes e/ou ovos foi observada entre filhos de mães sem atividade remunerada, tanto no primeiro quanto no segundo ano (Tabela 4). Desigualdades semelhantes foram identificadas para o indicador ZFV, mais frequente entre crianças de famílias mais pobres (Tabela 4).

**Tabela 3:** Razão de prevalência ajustada e respectivos intervalos com 95% de confiança para fatores associados aos indicadores diversidade mínima alimentar nos seguimentos de um e dois anos do estudo MINA-Brasil.

Variável	DMA Um ano (n = 767)^a^	DMA Dois anos (n = 829)^a^
	RP (IC95%)	RP (IC95%)
Cor da pele da mãe		
Branca	Ref.	-
Outras	0,89 (0,80–1,00)	-
Escolaridade materna		
≤ 9 anos	-	0,71 (0,60–0,83)^b^
Entre 10 e 12 anos	-	0,84 (0,75–0,94)^b^
> 12 anos	-	Ref.
Recebe Bolsa Família		
Não	-	1,11 (0,98–1,26)
Sim	-	Ref.
Mãe chefe de domicílio		
Não	-	Ref.
Sim	-	0,77 (0,64–0,92)^b^
Realiza atividade remunerada		
Não	0,87 (0,80–0,95)^b^	-
Sim	Ref	-

RP: razão de prevalência; IC95%: intervalo de confiança de 95%; DMA: diversidade mínima alimentar.

^a^ Totais em cada variável diferem devido à ausência de informação.

^b^ p < 0,05.

**Tabela 4:** Razão de prevalência ajustada e respectivos intervalos com 95% de confiança para fatores associados ao consumo de carnes e/ou ovos e zero consumo de frutas e vegetais nos seguimentos de um e dois anos do estudo MINA-Brasil.

Variável	Consumo de carnes e/ou ovos Um ano (n = 773)^a^	Consumo de carnes e/ou ovos Dois anos (n = 851)^a^
	RP (IC95%)	RP (IC95%)
Realiza atividade remunerada		
Não	0,88 (0,81–0,97)	0,93 (0,88–0,97)^b^
Sim	Ref.	Ref.
Insuficiência de vitamina A -criança		
Não	Ref.	-
Sim	0,80 (0,64–0,99)	-
**Variável**	**Consumo ZFV 1 ano (n = 761)^a^**	**Consumo ZFV 2 anos (n = 732)^a^**
	**RP (IC95%)**	**RP (IC95%)**
Escolaridade materna		
≤ 9 anos	-	1,56 (0,93–2,60)
Entre 10 e 12 anos	-	0,99 (0,60–1,63)
> 12 anos	-	Ref.
Índice de riqueza		
1º terço (menor)	4,89 (2,90–8,24)^b^	-
2º terço	2,47 (1,45–4,21)^b^	-
3º terço (maior)	Ref.	-

RP: razão de prevalência; IC95%: intervalo de confiança de 95%; ZFV: zero consumo de frutas e vegetais.

^a^ Totais em cada variável diferem devido à ausência de informação.

^b^ p < 0,05.

Em relação ao consumo de AUP, filhos de mães com maior escolaridade (> 12 anos) apresentaram menores frequências de consumo, enquanto aqueles cujas mães tinham menor escolaridade apresentaram maior consumo, tanto no primeiro quanto no segundo ano de idade (Tabela 5).

**Tabela 5:** Razão de prevalência ajustada e respectivos intervalos com 95% de confiança para fatores associados ao consumo de ultraprocessados com um e dois anos.

Variável	Consumo de AUP Um ano (n = 768)^a^	Consumo de AUP Dois anos (n = 829)^a^
	RP (IC95%)	RP (IC95%)
Idade materna		
< 19 anos	1,14 (0,98–1,32)	1,07 (0,99–1,16)
19 a 34 anos	1,07 (0,92–1,24)	1,04 (0,95–1,13)
≥ 35 anos	Ref.	Ref.
Cor da pele da mãe		
Branca	Ref.	-
Outras	0,93 (0,85–1,01)	-
Escolaridade materna		
≤ 9 anos	1,12 (0,98–1,27)	1,11 (1,04–1,20)^b^
Entre 10 e 12 anos	1,13 (1,01–1,27)^b^	1,09 (1,02–1,17)^b^
> 12 anos	Ref.	Ref.
Índice de riqueza		
1º terço (menor)	0,94 (0,85–1,04)	-
2º terço	1,02 (0,95–1,10)	-
3º terço (maior)	Ref.	-
Peso da criança ao nascer (n = 970)		
< 2.500 g	-	0,95 (0,87–1,04)
≥ 2.500 g a 3.999 g	-	Ref.
≥ 4.000 g	-	1,04 (0,99–1,09)
Anemia		
Não	Ref.	-
Sim	1,08 (1,02–1,15)^b^	-
Deficiência de vitamina A		
Não	Ref.	-
Sim	1,10 (1,03–1,17)^b^	-

RP: razão de prevalência; IC95%: intervalo de confiança de 95%; AUP: alimentos ultraprocessados.

^a^ Totais em cada variável diferem devido à ausência de informação.

^b^ p < 0,05.

A Figura mostra diferenças consistentes nos IQAC segundo índice de riqueza e escolaridade materna, em ambos os seguimentos. Para o DMA, observam-se maiores frequências entre crianças de famílias com melhores condições socioeconômicas, tanto em termos de riqueza quanto de escolaridade materna, com padrão semelhante nos dois seguimentos. Tendência semelhante foi observada para o consumo de carnes e/ou ovos, com maiores frequências entre crianças de famílias mais favorecidas. Em contraste, o consumo de AUP foi elevado em todos os estratos, embora ligeiramente menor entre filhos de mães com maior escolaridade. Por outro lado, o indicador ZFV foi mais frequente entre crianças de famílias com piores condições socioeconômicas, evidenciando desigualdades desfavoráveis nesses grupos. De modo geral, os *equiplots* indicam diferenças nos padrões alimentares infantis segundo condições socioeconômicas, mantidas ao longo do tempo.

**Figura:** Gráficos *equiplot* de quatro indicadores da qualidade da alimentação complementar estudados segundo terços do índice de riqueza e escolaridade materna nos seguimentos de um e dois anos da coorte MINA-Brasil.

## DISCUSSÃO

Os resultados deste estudo evidenciaram que os indicadores positivos de AC (DMA e consumo de carnes e/ou ovos) foram mais frequentes entre crianças de contextos socioeconômicos mais favoráveis, enquanto menores frequências foram observadas entre crianças de famílias mais pobres e cujas mães tinham menor escolaridade, não exerciam atividade remunerada ou eram chefes de domicílio. De maneira semelhante, os indicadores negativos de AC (consumo de AUP e ZFV) foram mais frequentes entre crianças de famílias com menor renda e cujas mães possuíam menor escolaridade.

A influência de características socioeconômicas na diversidade da dieta de crianças vem sendo observada em diferentes estudos, indicando que famílias com maior renda e maior poder de compra têm maior acesso a diferentes grupos de alimentos e, portanto, oferecem uma dieta mais diversa às crianças^5,19,20^. Embora nem todos os estudos encontrem associação entre renda familiar e consumo de carnes e/ou ovos^21^, evidências nacionais indicam maior consumo de alimentos frescos, incluindo carnes não processadas, entre crianças de famílias com maior poder aquisitivo^22^. Esses achados são consistentes com os resultados do presente estudo e sugerem que a ausência de renda materna pode contribuir para maior vulnerabilidade econômica no domicílio.

Dados do Enani 2019 apontaram a região Norte do Brasil com a menor prevalência de diversidade alimentar mínima entre crianças de seis a 23 meses (54,8%) em comparação com a média nacional (63,4%)^10^. Também foi observada menor prevalência desse indicador entre crianças cujas mães ou responsáveis apresentavam baixa escolaridade (zero a sete anos de estudo: 50,6%) e entre aquelas residentes em domicílios com insegurança alimentar moderada ou grave (52,6%). Esses achados reforçam a relação entre condições socioeconômicas adversas e piores padrões alimentares na infância, em consonância com os resultados aqui observados.

Observou-se que, quanto maior a escolaridade materna, menor o consumo de AUP pelas crianças. Esse padrão é consistente com a literatura e pode refletir diferenças socioeconômicas que influenciam o acesso, o conhecimento e as escolhas alimentares. Em um estudo de coorte conduzido no estado do Maranhão (n = 1.185), as crianças cujas mães tinham menos de oito anos de escolaridade apresentaram 37% mais chances de consumir AUP; entre aquelas com nove a 11 anos de estudo, a chance foi 25% maior, em comparação com filhos de mães com mais de 12 anos de ensino^23^. Resultados semelhantes foram observados no sul do Brasil em estudo transversal realizado em um Hospital terciário de Porto Alegre (n = 300) entre 2012 e 2013, observando associação inversa entre a escolaridade da mãe e o consumo de AUP entre crianças de quatro a 24 meses^24^. Resultados consistentes foram identificados pelo Enani 2019, o qual apontou menor consumo de AUP entre crianças cujas mães tinham maior escolaridade, em comparação àquelas com escolaridade inferior a sete anos ou entre oito e 11 anos^25^. Esse padrão pode refletir maior letramento em saúde entre mães com maior escolaridade, favorecendo escolhas alimentares mais saudáveis na infância^26^.

No presente estudo, o indicador ZFV foi mais frequente entre crianças de famílias com menor renda, evidenciando um padrão alimentar mais desfavorável nesses grupos. Esse achado é consistente com baixa frequência de consumo na população brasileira^27^, que apontam menor consumo desses alimentos entre populações de menor nível socioeconômico, possivelmente devido a limitações de acesso e ao maior custo de alimentos frescos. Dada a importância das frutas e hortaliças para a qualidade da dieta e para o estabelecimento de hábitos alimentares saudáveis na infância^28^, a ausência de consumo desses alimentos pode ter implicações para a saúde em fases posteriores da vida^30^.

Entre as limitações deste estudo, destaca-se que adaptações na operacionalização dos indicadores de AC impediram o seguimento integral da proposta da OMS. Em particular, a ausência de informação sobre alimentos ricos em vitamina A impediu sua inclusão no indicador de diversidade mínima da alimentação, o que pode limitar a comparabilidade com outros estudos, embora sua estrutura geral tenha sido preservada. O recordatório de 24 horas está sujeito a erro de mensuração e viés de memória, podendo não refletir o consumo habitual individual, e não foi possível ajustar a variabilidade intraindividual, dado o uso de medidas em momentos distintos (um e dois anos). O indicador de AUP foi baseado na classificação NOVA, o que pode limitar comparações com o indicador da OMS, embora aumente a especificidade para alimentos associados a piores desfechos. As análises apresentam caráter transversal, limitando inferência causal, e as perdas de seguimento podem ter introduzido viés de seleção, ainda que com pouca evidência de diferenças relevantes entre participantes acompanhados e não acompanhados.

Os achados deste estudo apontam para diferenças importantes no padrão da AC entre crianças da Amazônia Ocidental Brasileira, refletindo o impacto das condições socioeconômicas sobre a qualidade da dieta infantil. Esses resultados reforçam a necessidade de estratégias intersetoriais voltadas à promoção da alimentação saudável na infância visando reduzir desigualdades e promover ambientes alimentares mais saudáveis.

## Data Availability

Os dados estão disponíveis mediante solicitação ao autor correspondente.
